# Effect of Meditation on Cognitive Functions in Context of Aging and Neurodegenerative Diseases

**DOI:** 10.3389/fnbeh.2014.00017

**Published:** 2014-01-27

**Authors:** Rafał Marciniak, Katerina Sheardova, Pavla Čermáková, Daniel Hudeček, Rastislav Šumec, Jakub Hort

**Affiliations:** ^1^International Clinical Research Center, St. Anne’s University Hospital Brno, Brno, Czech Republic; ^2^Alzheimer’s Disease Research Center, Department of Neurobiology, Care Sciences and Society, Karolinska Institutet, Stockholm, Sweden; ^3^Memory Disorders Clinic, Department of Neurology, 2nd Faculty of Medicine, Charles University in Prague and University Hospital Motol, Prague, Czech Republic

**Keywords:** meditation, cognition, dementia, aging, neurodegenerative diseases, Alzheimer’s disease, mild cognitive impairment, elderly

## Abstract

Effect of different meditation practices on various aspects of mental and physical health is receiving growing attention. The present paper reviews evidence on the effects of several mediation practices on cognitive functions in the context of aging and neurodegenerative diseases. The effect of meditation in this area is still poorly explored. Seven studies were detected through the databases search, which explores the effect of meditation on attention, memory, executive functions, and other miscellaneous measures of cognition in a sample of older people and people suffering from neurodegenerative diseases. Overall, reviewed studies suggested a positive effect of meditation techniques, particularly in the area of attention, as well as memory, verbal fluency, and cognitive flexibility. These findings are discussed in the context of MRI studies suggesting structural correlates of the effects. Meditation can be a potentially suitable non-pharmacological intervention aimed at the prevention of cognitive decline in the elderly. However, the conclusions of these studies are limited by their methodological flaws and differences of various types of meditation techniques. Further research in this direction could help to verify the validity of the findings and clarify the problematic aspects.

## Introduction

Increasing age of the world’s population leads to a high number of people suffering from dementia. Alzheimer’s Disease International association estimated that there are nearly 36 million people suffering from dementia. The number is expected to double every 20 years, therefore 66 million people could be affected by dementia in 2030 (Prince et al., 2013). This highlights the need for an appropriate therapy for patients with dementia, which is based both on pharmacotherapy and non-pharmacological interventions.

One kind of non-pharmacological approach is represented by cognitive training or stimulation aimed at impacting cognitive functions, most commonly memory, attention, orientation, or language. However, the effects of such interventions are not consistent. They seem to be more efficient in motivated patients in early or intermediate stages of dementia (Hulme et al., 2010; Olazaran et al., 2010). Other non-pharmacological ways include behavioral interventions aimed at improving skills in activities of daily living (ADL) or management of stimuli exposed to patients. Patients are encouraged to engage in appropriate physical activity and their emotional health is supported individually or in the form of group sessions. Attention is paid to the reduction of anxiety or sleep disorders. Other options of intervention with a positive impact on multiple domains (cognition, emotivity, ADL) are represented by ergotherapy, reminiscensce therapy, art therapy, relaxation, movement therapy, or musicotherapy (Gauthier et al., 2010; Olazaran et al., 2010). An important aspect of non-pharmacological approaches is the attempt to postpone the institutionalization of patients and the support of caregivers (education, self-supporting groups) in order to reduce a great burden to which they are exposed in later stages of the disease (Chien et al., 2011).

Other possibilities of non-pharmacological interventions are based on various meditation techniques. The impact of meditation on human health has been recently a subject of great scientific interest. The effect of these techniques has been studied from different perspectives (depression, anxiety disorders, eating disorders, addictions, and disorders caused by the use of psychoactive drugs) (Ospina et al., 2007; Balaji et al., 2012; Khanna and Greeson, 2013; Lakhan and Schofield, 2013). The impact of meditation on stress reduction, the prevention of psychosomatic disorders, blood pressure, and other cardiovascular diseases is a subject of several studies as well (Barnes et al., 2001; Grossman et al., 2004). Meditation can help with chronic pain and musculoskeletal disorders, respiratory diseases, and dermatological problems. It may be beneficial as a support of the immune system or as a symptomatic treatment of cancer (Ospina et al., 2007). Recently, there have been studies on the effect of meditation techniques on cognitive skills, which are reviewed in this paper in a specific context of aging and neurodegenerative diseases. Meditation techniques are considered to be specific cognitively stimulating activities. The effect of meditation on cognition is studied directly as well as from the perspective of the reduction of depressive symptoms and anxiety. There is a growing interest in meditation as one of the potential strategies for the prevention of Alzheimer’s disease (Horrigan, 2007).

## Meditation

Even though scientists have been investigating meditation for a long time, there has not been consensus on its definition. Diversity in the range of possible definitions reflects the vast number of different methods of meditation. Western definitions emphasize that meditation is a self-regulatory technique focused on maintaining one’s attention. However, in the spiritual tradition, meditation is perceived as a tool for spiritual development, the growth of inner peace, concentration, positive emotions, such as love and happiness, and on reduction of negative emotions, such as fear and anger. Walsh and Shapiro (2006) integrate those two views and propose a new definition. It characterizes meditation as a group of self-regulatory techniques focused on maintaining attention and awareness. The main goal is to achieve a greater rate of well-being, serenity, and concentration through the enhancement of control over spiritual processes. This definition distinguishes meditation from other methods, for example hypnosis, imagination, or psychotherapy. These techniques are not based on development of awareness or attention, but they rather focus on changing mental content of thoughts, images, and emotions.

Walsh and Shapiro (2006) suggest a classification of meditation according (1) to its area of interest: there are techniques, which primarily focus on a single object, such as breath or sounds. They are known as concentration meditations. Another type is represented by meditation, which aspires to gain open attention, containing more objects at once or selected in a consecutive order. This type is called awareness or open meditation. In addition, we can divide meditation techniques according to its relation to cognitive processes (thoughts, images) (2). This classification is consistent with the categories proposed by Lutz et al. (2008), who speaks about openly monitoring meditation (open monitoring, OM) and meditation with focused attention (focused attention, FA). The third type of classification relies on the targets (3). While some practices focus on supporting a general mental development and the state of well-being, others concentrate primarily on the growth of specific mental qualities, such as concentration, love, or wisdom. The most scientifically exploited techniques are described thoroughly below.

### Mindfulness

One of the most researched meditation techniques is based on the concept of mindfulness (in Pāli language *Satí*). Traditionally this method has its origin in Buddhist meditation of mindfulness and insight (in Pāli language “*satipatthana-vipassana*”). Mindfulness practice includes a number of meditational techniques, such as activities focused on breath and physical awareness or using metaphors enlightening the essence of mindfulness. All these techniques have a common goal, which is expanding a subject’s mindfulness – i.e., the ability to focus on the present moment and to perceive without any judgment or choice current internal or external impulses, which are emerging at a given moment of consciousness. Mindfulness thus allows one to stay “above” the particular content of thoughts, emotions, or imaginations and enables one to become aware of the process of consciousness itself (Kabat-Zinn, 2005). Mindfulness allows one’s active approach, which can alter current categories and distinctions through focusing on new impulses, which would otherwise remain unconsciously unnoticed. This conscious processing of impulses impacts a person’s behavior and supports a change of habitual behavioral patterns (Langer, 1989).

Personal experience of many western psychologists leads them to establish meditational techniques as a part of their psychotherapeutic praxis, in which they use the techniques based on mindfulness very frequently. There are many psychotherapeutic schools and approaches, which use the techniques based on the concept of mindfulness, for example, Gestalt therapy or Morit’s therapy. There are several new areas combining a mindfulness approach with cognitively behavioral therapy, such as mindfulness-based cognitive therapy, dialectical behavior therapy, and acceptance and commitment therapy (Germer et al., 2005).

#### Zen meditation

Zen meditation is often classified as a meditational technique based on fundamentals of mindfulness. It comes from Zen Buddhism, Mahayan Buddhism’s offshoot, which originated in the fifth century in China. It is performed sitting with legs crossed (lotus position) and the meditating person tries to maintain straight position of the body and a regular speed of breathing. On the mental level, they focus on their breath while their mind is open to emerging spiritual processes and contents, which they neither judge, conceptualize nor evolve. There upon moments of completely content-free consciousness occur (Pagnoni and Cekic, 2007).

### Transcendental meditation

Transcendental meditation represents another frequently used scientific method. It was developed by Maharishi Mahesh Yogi in the second half of the twentieth century, but it is based on ancient Indian Vedic tradition. This practice is based on the repetition of mantra for 15–20 min twice a day with closed eyes. Mantras in other words are sounds or simple sentences usually in Sanskrit facilitating the process of “inlaying” of attention. Attention is paid on inner psychological processes with the aim of overcoming even the mildest forms of thinking and to discover the source of thoughts, which is felt as a moment of pure consciousness, absolutely free of any content (Forem, 2012).

### Vihangam yoga

Another method, which has been investigated by researchers is Vihangam yoga. Its roots arise from the teaching of Sadguru Sadafaldeo Ji Maharaj. In theory and practice, it relates to the Indian Vedic tradition. The practice of this meditation is divided into five levels, but in scientific studies the most examined is the first one. In the first level, the meditating person tries through the training of concentration (for example, by repetition of mantra) to develop conscious reflexion, the ability to observe his mind’s own tendencies. This helps to get better orientation in one’s own inner world and to take better control of it. This state of mind allows subjective feelings of harmony and satisfaction.

### Kirtan Kirya

Experiments with the method of Kirtan Kirya are often performed in the context of neurodegenerative diseases. This technique originates in the tradition of Kundalini yoga school. The technique itself is based on repetition of sounds “sa ta na ma,” loudly, in a whisper and silently in 2 min periods. Meanwhile the meditator touches the rest of the fingers with their thumb. According to Kundalini yoga, 84 acupuncture points are being stimulated while performing this technique. This leads to a positive bio-chemical transformation in the brain. From a neuropsychological point of view the effect of this method is explained as the activation of the brain areas associated with attention and exclusive functions (frontal area, cingulate cortex), which takes places during the meditation (Newberg et al., 2010a).

### Relaxation

The effect of meditation is often compared with the effect of relaxation. Relaxation can be understood as a reduction of neurophysiological agitation (Benson et al., 1975). Meditation as a certain type of mental exercise can be included in such a broadly defined framework. But in the context of studies mentioned below, relaxation is a physical exercise focused on releasing muscles, which reduces somatic stress (Schwartz et al., 1978), or a mental relaxation, which does not cooperate with conscious focus of attention, but only instructs the subject to sit calmly with closed eyes (Alexander et al., 1989). However meditation has some additional features and can be defined as a group of self-regulatory techniques focused on maintaining attention and awareness, where the main goal is developing voluntary control over mental processes to achieve a higher overall level of well-being and also to achieve peace and concentration (Walsh and Shapiro, 2006).

## Research

There is an increasing amount of literature suggesting that there are many areas, which can be influenced by meditation. The most commonly studied topics include physiological, psychiatrical, and psychological conditions (e.g., anxiety, depression, quality of life, or impact on ADL) or a general medical condition (Ospina et al., 2007). Another subject of research is the effect of meditation techniques on cognition and neuropsychological functions. Various types of mindfulness meditation seem to positively influence cognitive functions. A review by Chiesa et al. (2011) suggests a significant improvement of selective and executive attention in early stages of meditation, which aims at cultivating focused attention. Non-focused, long-term attention can be improved during following stages of meditation, which are characterized by non-judgmental observation of external and internal stimuli. Besides, this technique can increase the capacity of working memory and several executive functions. However, many studies are biased due to methodological mistakes and connections of researchers with institutions which propagate a specific type of meditation.

The studies in this review were selected through a search in scientific databases (PubMed, SpringerLink, JSTOR, EBSCO, ISI, ScienceDirect, SCOPUS, Wiley, ProQuest) by using relevant keywords (meditation, neurodegenerative disorders, dementia, Alzheimer’s disease, aging, etc.). Studies investigating the effect of meditation on cognition in which aging people and people with neurodegenerative diseases were included.

### Meditation as a preventive strategy against Alzheimer’s disease

Alzheimer’s disease is the most common cause of dementia and is strongly related to age (Wallin et al., 2013). Other risk factors include family history, hypertension and hypotension, high levels of cholesterol, low physical activity, obesity, low level of education, and the presence of ApoE4 (Kivipelto et al., 2001, 2005; Huang et al., 2004). At least five studies have been published during the past 2 years suggesting that the incidence of dementia may have decreased over the last two decades (Rocca et al., 2011; Schrijvers et al., 2012; Christensen et al., 2013; Matthews et al., 2013; Qiu et al., 2013). The mortality improvements are generally attributed to better awareness and successful management of its risk factors. It was estimated that delaying the onset of AD by a mere year would yield nine million fewer cases by 2050 (Brookmeyer et al., 2007). The prevention of dementia may to be more effective than current pharmacological treatment (Forette et al., 1998; Khachaturian et al., 2006). A general agreement concerning cognitive decline at advanced age motivates many scientists to search for new preventive strategies to maintain cognitive functions until the end of life (Salthouse, 2011). There is a growing evidence that meditation can serve as a potential tool for the prevention of Alzheimer’s disease (Horrigan, 2007).

**Figure 1:**
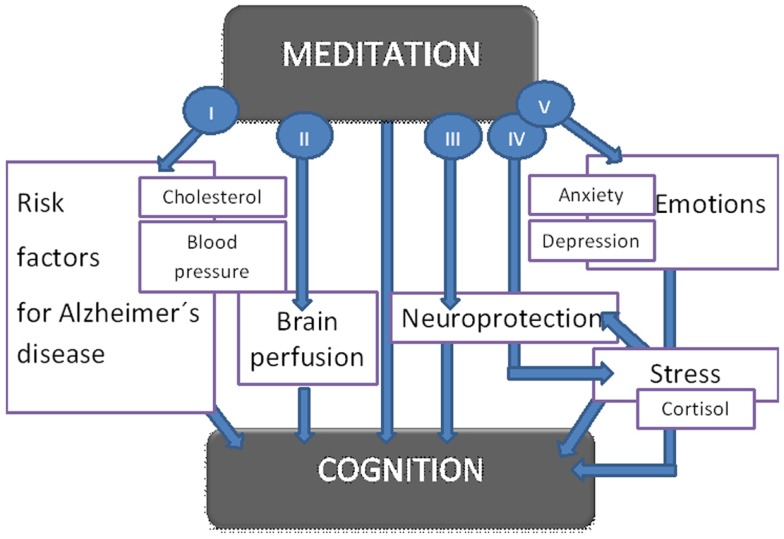
**Suggested influence of meditation on cognitive functions**. The figure shows proposed ways how meditation impacts cognitive functions. The effect of meditation on cognition is both direct and indirect (I–V): meditation positively influences hypercholesterolemia and hypertension which represent risk factors for Alzheimer’s disease (I). Further it increases cerebral blood flow (II) and has a protective effect on the cortical thickness (III). Meditation further reduces stress (IV), anxiety, and depression (V). All these mechanisms lead to better cognitive functions.

It has been revealed that meditation can influence risk factors of Alzheimer’s disease such as hypertension (Anderson et al., 2008) and high levels of cholesterol (Walton et al., 2004; Khatri et al., 2007). Besides, the impact of meditation on the cerebral blood flow (Newberg et al., 2001, 2010b; Khalsa et al., 2009) could play a role in Alzheimer’s disease as well (Roher et al., 2012) (Figure 1).

### Meditation and cognition in aging population

Research on preventive strategies against abnormal aging usually focuses on life style, in particular on physical activity and healthy diet. Only a few studies emphasize the potentially protective effect of meditation on cognition.

One of the first studies on the effect of meditation in elderly was performed by Alexander et al. (1989). The population was represented by 73 seniors (mean age 81 years) who were randomly divided into three groups based on the meditation technique they underwent. A control group without any intervention was also included. Seniors were trained in the following meditation techniques: transcendental meditation, mindfulness (*mindfulness training in active distinction making*) and a relaxation program which they performed twice a day for 20 min a period of 12 weeks. This study examined the effect of the intervention on cognitive flexibility (*Stroop’s test*), memory (*Associate Learning subtest*), and verbal fluency of the elderly (*Word Fluency Scale, Overlearned Verbal Task*). These were measured before and immediately after the meditation, after 18 months and finally after 36 months. The results suggest a strong improvement in measured variables in the group of subjects using transcendental meditation, followed by mindfulness. Worse results were shown in the control group and in the group with the relaxation program. In addition, testing after 3 years revealed 100% maintained effect in persons using transcendental meditation and 87.5% in those within the mindfulness program. Other groups had lower scores (65 and 75%). Even though the described study suggested a potentially positive impact of meditation on cognitive decline in the elderly, no other studies had been performed until recently.

A study by Pagnoni and Cekic (2007) examined in a case–control study (*n* = 26) the effect of regular Zen meditation on the decrease in gray matter thickness and ability to solve tasks demanding focused attention. While the study showed age-related decrease in gray matter thickness in the case of 13 subjects in the control group (without any intervention), there was no correlation between any studied variable and age in the case of the 13 subjects who regularly meditated. The effect of meditation on gray matter was the most significant in putamen, which is a structure involved in attention processing.

Another case–control study (van Leeuwen et al., 2009) compared long-term meditating seniors using mindfulness with two groups of control participants, who had never engaged in any meditation practice. In the first one, there were people of the same age. Younger people belonged to the second control group. The groups were compared on the basis of their performance in *attentional blink*. The results of meditating group were significantly better compared to the age-matched control group. Furthermore, they performed better also in comparison with the control group consisting of young people, even though several studies have proved that attention ability decreases with age (Maciokas and Crognale, 2003; Georgiou-Karistianis et al., 2007).

A recent cross-sectional study (Prakash et al., 2012) compared cognitive skills between long-term meditating and non-meditating seniors. Twenty seniors with more than 10 years of experience with Vihanam yoga meditation underwent a battery of tests on short-term memory, psychomotoric tempo, attention, and executive functions (*Digit Span test*, *Stroop*’*s test*, *Trail Making test*, *Letter Cancelation Task*, *Digit symbol substitution test*, and *Rule Shift Card Test*). The results were compared with the performance of non-meditating seniors. Meditating seniors showed significantly better results in all attentional tests. The study revealed that long-term meditation of Vihanagam yoga impacted positively on the extent of attention, the speed of processing, the ability of attentional shift, and performance in tests using distracting factors.

### Imaging studies

MRI studies provide an interesting insight into morphological changes of the brain resulting from meditation. Even though they do not investigate elderly population, they reveal structural changes in several regions, such as increased cortical thickness. Most frequently reported are structural alterations in anterior cingulate cortex, superior and inferior frontal cortex, and prefrontal cortex. These regions are involved in attention, perceiving internal experience, sensory processing, and executive functions. Some studies report increased volume of hippocampi, which are important for memory. For an overview of MRI studies on meditation and their findings, see Table 1. Similar regions of activation are reported from functional imaging studies. SPECT performed during meditation showed increased regional cerebral blood flow in the prefrontal cortex, superior frontal, and cingulate cortex, and the right temporal lobe (Wang et al., 2011).

**Table 1:** **List of brain imaging studies using MRI**.

Study	Intervention	*n*	Mean age ± SD	Experience with meditation	Loci with increased cortical thickness	Interpretation
Lazar et al. (2005)	Various	20	38.2	9.1 ± 7.1 years, 6.2 ± 4 h per week	Anterior insula, parts of frontal lobe, auditory cortex in temporal lobe	Somato-sensory, auditory, and interceptive processes
Pagnoni and Cekic (2007)	Zen	13	37.2 ± 6.9	>3 years per day	Putamen	Attention
Holzel et al. (2008)	Vipassana	20	34.1 ± 4.7	8.6 years, 2 h daily	Anterior insula, right hippocampus, left inferior temporal gyrus	Anterior insula – awareness of internal experience
Vestergaard-Poulsen et al. (2009)	Tibetan buddhism	10	55 ± 6.2	16.5 ± 5.1 years	Medulla oblongata, anterior cerebellum, superior, and inferior frontal gyrus	Breath control, resistance to stress, attention, calmness
Luders et al. (2009)	Various	22	53 ± 11.5	24 ± 12 years	Orbito-frontal cortex, right talamus, left inferior temporal gyrus	Regulation of emotions and sensory functions
Grant et al. (2010)	Zen	17	37.6 ± 10.9	>1000 h	Anterior cingulate cortex, secondary somato-sensory cortex	Anterior cingulate cortex – adaptive control of behavior
Holzel et al. (2011)	MBSR	16	39 ± 4	0	Left hippocampus, posterior cingulate cortex, temporo-parietal junction, cerebellum	Learning, memory, regulation of emotions, empathy
Luders et al. (2013b)	Various	50	51.4 ± 12.8	20 years	Hippocampus, especially subiculum	Subiculum – regulation of stress
Grant et al. (2013)	Zen	18	37.1 ± 10.9	>1000 h	Cingulo-fronto-parietal network	Attention

**n*, number of subjects, SD, standard deviation, MBSR, mindfulness-based stress reduction, IBMT, integrative body mind training*.

Moreover, in the study by Lazar et al. (2005), the biggest effect of mindfulness meditation on the thickness of the prefrontal cortex was surprisingly found in older participants. This suggests that meditation can have a compensatory effect on the decrease of cortical thickness related to aging. The increased cortical thickness found in meditators can be explained by several mechanisms: neuronal arborization, multiplication of glial cells, or formation of vessels (Lazar et al., 2005). This also implies that meditation could potentially lead to neuroregeneration.

Study by Luders et al. (2011) explores the fractional anisotropy for 20 different fiber tracts on sample of long-term meditators (Shamatha, Vipassana, and Zazen) and controls (*n* = 54). Results showed stronger structural connections in meditators compared to controls throughout the brain in large projection pathways, commissural pathways, and association pathways. Although fractional anisotropy and age were negatively correlated in both groups, regression lines of age-related decline in meditators were much less marked than in controls.

### Interventions studies and neurodegenerative diseases

Research of meditation from the perspective of neurodegenerative disorders is still in its infancy. It includes studies on the effect of meditation on well-being of caregivers (Waelde et al., 2004; Lavretsky et al., 2013) as well as on patients suffering from dementia. As Newberg et al. (2013) summarized, current knowledge about meditation, memory, and attention supports the application of meditation techniques in patients with neurodegenerative diseases. Below are presented concrete studies investigating the effect of meditation on cognition on a sample of patients with neurodegenerative diseases.

An article by Newberg et al. (2010b) examined the effect of an 8-week meditation program using a simple method of Kirtan Kirya. The control group was listening to music instead of performing meditation. Fifteen seniors with age-related cognitive impairment (*n* = 7), mild cognitive impairment (*n* = 5), and Alzheimer’s disease (*n* = 3) were included in the study. Cerebral blood flow and performance in cognitive tests were examined. The effect of the 8-week long meditation program showed a significant increase in cerebral perfusion in prefrontal, parietal, and auditory cortex. The results of neuropsychological tests showed an improvement in verbal fluency, part B in *Trail making test* (test on working memory and attention) and logical memory in the meditating group. Most of the participants also expressed a significant subjective improvement in cognitive functions.

Similar research by Moss et al. (2012) studied the effect of an 8-week meditation program in Kirtan Kirya technique. They measured the effect of this program on depression and anxiety. Cerebral perfusion, level of spirituality, cognitive functions in categorical fluency, *Trail making test* (part B), and logical memory was also examined. The participants consisted of seniors with impaired memory (*n* = 7), mild cognitive impairment (*n* = 5), and Alzheimer’s disease (*n* = 3). The control group listened to music in this study as well. The results in the meditating group revealed a significant improvement in depression, anxiety, internal tension, and fatigue. Increased cerebral perfusion in the frontal lobe and right parietal lobe has also been found. However, in this research, there was no significant effect of meditation on cognitive functions. Interestingly, there were no significant changes in spirituality scores [index of core spiritual experiences (INSPIRIT), the purpose in life scale, the mysticism scale, the quest scale, and mindful attention awareness scale] over the 8-week period.

A recent study by Innes et al. (2012) examined the effect of Kirtan Kirya on stress, quality of sleep, mood, sympathetic activation, and memory functions in adults suffering from cognitive decline. The effect was also studied on their caregivers. Six patients in early stages of Alzheimer’s disease and their caregivers were tested before and after undergoing an 8-week meditation program. The participants showed a significant improvement in retrospective memory (tested by *Memory Functioning Questionnaire*) and also in other measured variables, such as stress, mood disorders, quality of sleep, and blood pressure.

## Discussion

Insufficient amount of research on the effect of meditation on age-related cognitive decline and neurodegenerative diseases makes any generalization of the results very difficult. However, the reviewed studies suggest a positive effect of various meditation techniques on particular cognitive functions. There is evidence that meditation enhances attention (Pagnoni and Cekic, 2007; van Leeuwen et al., 2009; Prakash et al., 2012), improves verbal fluency (Alexander et al., 1989; Newberg et al., 2010a), memory (Alexander et al., 1989; Newberg et al., 2010a; Innes et al., 2012), and cognitive flexibility (Alexander et al., 1989; Newberg et al., 2010a).

Results mentioned above suggest a possible explanation of the impact of meditation on processes in the human brain (see Figure 1). The mechanisms include increased cerebral perfusion in prefrontal, parietal and auditory cortex (Newberg et al., 2010a), a protective effect on gray matter thickness (Pagnoni and Cekic, 2007), and enhancing of the function of areas involved in attention (Lazar et al., 2005). In addition, meditation can potentially enhance the power of cognitive circuits and increase cognitive capacity (Xiong and Doraiswamy, 2009). Moreover, it can improve myelination or restructuralization of white-matter tracts in the involved areas such as anterior corona radiate associated with the anterior cingulate cortex (Tang et al., 2010).

Another explanation of the neuroprotective effect of meditation can be the decrease in cortisol level (Jacobs et al., 2013; Turakitwanakan et al., 2013) caused by stress, which may be related to a higher hippocampal volume in meditators (Luders et al., 2013a). Epel et al. (2009) emphasize the correlation between the maintenance of the length of telomeres and decreased cognitive stress and tension due to meditation (Jacobs et al., 2011; Hoge et al., 2013) Meditation can positively impact dyslipidemia and oxidative stress, which further decreases the risk of vascular diseases of the brain as well as Alzheimer’s disease (Reitz, 2013).

From a psychological point of view, the effects on cognitive functions can be explained by enhancing the ability of mindfulness (in the case of mindfulness meditation). Mindfulness enables a non-judgmental reflexion of processes happening in consciousness “beyond” concrete contents of thoughts and feelings. This leads to experiencing a relativity and transient nature of these contents, which can (from the long-term point of view) lead to weakening of affective power of these perceptions in consciousness (e.g., as in anxiety) and enlarging the capacity for focused processing. This ability can lead to improvement in attentional and working memory tasks. Similarly, we can look at the effect of meditation based on mantra repetition (transcendental meditation, Kirtan Kirya, etc.). This method aims at releasing consciousness from constantly appearing language based thoughts. Short-lasting experience of the relief of consciousness from its contents (thoughts and feelings) can lead to understanding of their relativity and transience (Alexander et al., 1989). It is necessary to emphasize the potential of various meditation techniques in the enhancement of flexibility, contrasts with its decrease during aging. This is related to higher perception of new stimuli, which are not in line with old cognitive schemes and habitual behavioral patterns. This supports adaptive behavior based on one’s own decisions (Alexander et al., 1989; Langer, 1989). An important aspect, which is enhanced by all the mentioned types of meditation, is self-reflexion and cultivation of the ability to deal with one’s own mental processes. Such skills can contribute to decreased depressivity and anxiety, which have negative effect on cognitive functions (Beaudreau and O’Hara, 2008).

The positive potential of meditation, which is suggested from research reported here, has to be related to the limitation of these studies, which are often preliminary capture. Many papers included too few subjects, control groups were missing or the research has been performed by institutions supporting a special type of meditation. In many studies, the effect of meditation on cognition was often measured by many cognitive tests, authors of the studies pointed out significant positive changes, but there should be also emphasized fact, that in some tests, the effect on specific cognitive functions has not been proved, as you can see in Table 2. For example, in study of Moss et al. has not been proved significant effect of meditation on cognitive functions, only on the other measured variables.

**Table 2:** **List of studies investigating the effect of meditation on cognition on a sample of elderly people and people with neurodegenerative diseases**.

Study	Participants	Significant effect oncognitive functions	Memory	Attention	Executive functions^a^
Alexander et al. (1989)	Elderly		Yes	Yes	Yes
Pagnoni and Cekic (2007)	Elderly		–	Yes	–
van Leeuwen et al. (2009)	Elderly		–	Yes	–
Newberg et al. (2010a)	Elderly, MCI, Alzheimer’s disease		Yes	Yes	Yes
Newberg et al. (2013)	Elderly, MCI, Alzheimer’s disease		No	No	No
Grant et al. (2010)	MCI, Alzheimer’s disease, caregivers		Yes	–	–

*^a^Into executive functions are assigned cognitive flexibility and verbal fluency*.

An attempt to generalize such results is in contrast with the fact that meditation comprises a heterogeneous group of practices. It is becoming evident that in the context of neurodegenerative disorders there is a lack of studies using methods other than Kirtan Kriya. The key tasks for future studies will be identification of a potential common element of different meditation techniques, establishment of a valid tool to measure meditation techniques, better control of life style factors, genetics, and eating habits, applying findings on larger randomized population samples and finding out whether the results observed in highly experienced meditation practitioners can be found in a wider population.

Various types of meditation were traditionally established among religious systems, which related a close connection between meditation and the spiritual part of human beings. It is becoming evident that, despite the loss of intellectual and memory skills, patients with Alzheimer’s disease often maintain spiritual consciousness and intuition (Dopson, 2005). Such patients still manage, despite a significant memory loss, to learn to use simple meditation techniques. Involving patients with Alzheimer’s disease in activities, such as prayers or meditation, can positively impact on the quality of their life, spiritual well-being, feelings of self-value and belonging (Lindberg, 2005).They can also decrease anxiety related to this disease and serve as a potentially useful intervention for improving negative symptoms and behavior. Studies by Kaufman et al. (2007) emphasize slower cognitive decline and progression of Alzheimer’s disease in patients with higher level of spirituality or personal religious practice, however, another study (Levin et al., 1994) did not show that regular visits to church slow down the progression of Alzheimer’s disease. These findings imply an alternative explanation of the impact of meditation, which is by enhancing a spiritual dimension of the elderly. Even though researchers suggest a different effect of secularly and spiritually aimed meditation on the health and well-being of meditators (Wachholtz and Pargament, 2005), spirituality has been among measured variables in only one of the reported studies (Moss et al., 2012). However, there was no significant association with the effect of meditation, therefore the question of the relationship between meditation and spirituality remains open to further research.

## Conclusion

There is an increasing amount of literature suggesting a positive impact of meditation on physical and psychological health. Recently, there have been studies on the influence of meditation on cognitive functions in the context of aging and neurodegenerative diseases. The results imply a positive effect especially on attention, memory, verbal fluency, and cognitive flexibility. Meditation can represent an appropriate non-pharmacological intervention aiming at the prevention of cognitive decline in the elderly. Conclusions of such studies are limited due to their methodological problems and differences among various meditation techniques. Further research in this area could help to confirm the validity of recent results and clarify problematical aspects.

## Conflict of Interest Statement

The authors declare that the research was conducted in the absence of any commercial or financial relationships that could be construed as a potential conflict of interest.
